# Stakeholder engagement champions: a locally-driven model for building impactful and sustainable relationships in global health research

**DOI:** 10.7189/jogh.15.03026

**Published:** 2025-06-06

**Authors:** Tracy Jackson, Genevie Fernandes, Siân Williams, Jayakayatri Jeevajothi Nathan, Meiko Makita, RESPIRE Collaboration

**Affiliations:** 1 Usher Institute, University of Edinburgh, Edinburgh, Scotland, UK; 2 International Primary Care Respiratory Group, Edinburgh, Scotland, UK; 3 Faculty of Medicine, Department of Primary Care, University of Malaya, Kuala Lumpur, Malaysia; 4 School of Health Sciences, University of Dundee, Dundee, UK

## Abstract

Stakeholder engagement is increasingly required by global health funders as a means to enhance research impact and bridge the gap between knowledge generation and its application in local health systems. However, global health researchers often face structural and operational barriers that limit meaningful stakeholder engagement. We present a practical model developed by a global respiratory health research programme implemented in four low- and middle-income countries (LMICs) – Bangladesh, India, Malaysia, and Pakistan. The model centred on designating in-country stakeholder engagement champions – locally based professionals with strong communication skills and contextual understanding of the health system and stakeholders. These champions were supported through mentorship, peer exchange, and capacity-building activities delivered by a central community and stakeholder engagement platform comprising experts and researchers. The champions had autonomy to design context-specific engagement strategies, allocate resources, and lead interactions with stakeholders throughout the research lifecycle. This decentralised approach enabled tailored engagement, fostered south-south learning, and created leadership opportunities for LMIC researchers. Despite successes, challenges included managing power imbalances, limited institutional capacity, and increased workloads. The model offers a promising approach for advancing equitable partnerships and local leadership in global health research, aligning with broader efforts to decolonise global health and promote meaningful, context-driven stakeholder engagement.

In the context of global health research, stakeholder engagement is an iterative process where researchers actively seek the knowledge and experience of individuals or groups that are interested in, or impacted by, a disease, a health condition or an intervention. In doing so, they enable these stakeholders, including patients, public, community leaders, health workers, health care providers, and policy makers, to support, contribute, collaborate or partner in making effective decisions around research and translation [1]. Stakeholder engagement has become a standard expectation of global health research funders as it can align research studies with national and local health priorities and policy agendas, and increase support and buy-in for adopting and scaling up effective interventions, [2–4] thereby bridging the gap between knowledge production and its use within health systems [5].

Whilst the principle is accepted, several operational and structural barriers can impede global health researchers from practising stakeholder engagement and realising its full potential. Stakeholder engagement may not be adequately understood or considered significant in the broader context of the research study, leading to inadequate allocation of time or resources. Research teams may lack dedicated, skilled professionals to plan and implement tailored engagement and communication strategies. Constrained study timelines can further exacerbate these barriers. Concerns have also arisen about the tokenistic involvement of stakeholders in health research, particularly patients, community and public members [6,7].

Stakeholder engagement can also be compromised due to power imbalances between researchers in high-income countries and those in low- and middle-income countries (LMICs) and even between researchers and stakeholders within LMICs [8,9]. These imbalances can be heightened by the disproportionate decision-making powers of HIC experts, often with minimal lived experience of working in the study setting or living with a specific condition, whereas there is limited participation and autonomy of LMIC experts and local stakeholders in governance structures and leadership roles [10]. Merely shifting this imbalance around knowledge and leadership is not enough; LMIC partners need capacity and infrastructure to practice meaningful stakeholder engagement.

Even when researchers are committed to involving stakeholders, funding can be a major barrier. Researchers receive funding only after being awarded a grant [6]. Therefore, without any discretionary budget, stakeholders cannot be reimbursed for their time spent on any of the pre-award activities, including prioritisation of research questions and development of the research proposal. As a result, researchers may miss out on valuable stakeholder input, enabling them to align the study with local priorities and needs, or they may rely on the same stakeholders consulted in previous studies. When stakeholders choose to contribute to pre-award activities without any reimbursement for their time, researchers still need to cover expenses related to in-person meetings (*e.g.* venue hire, food and drink, and travel fares); often considered essential in LMICs cultures [11]. Further, inadequate funding and a lack of flexibility in budgetary reallocation, especially during unexpected events, make it difficult to engage meaningfully with stakeholders and sustain this after the study has ended.

In this article, we reflect on how we aimed to address these challenges above in realising meaningful stakeholder engagement in the Global Health Research Unit on Respiratory Health (RESPIRE). We describe our novel approach, share the lessons learned and challenges faced, and offer insights for global health researchers and practitioners that could be adapted to other contexts.

## RESPIRE: ADVANCING RESPIRATORY HEALTH RESEARCH IN ASIA

Funded by the UK National Institute of Health and Care Research between 2017–22, RESPIRE was a global respiratory health research collaboration spanning across Asia with 10 research partner organisations in Bangladesh, India, Malaysia and Pakistan working with the University of Edinburgh and the International Primary Care Respiratory Group in the UK [12]. The RESPIRE partnership resulted in 52 research studies mapping the burden of respiratory diseases and testing interventions to reduce this burden, strengthened the capacity of researchers, trained more than 500 frontline health workers and health care providers, and advocated for delivering effective interventions in local health systems in the four countries [13]. Stakeholder engagement was considered a critical component in achieving these outcomes.

**Figure 1:**
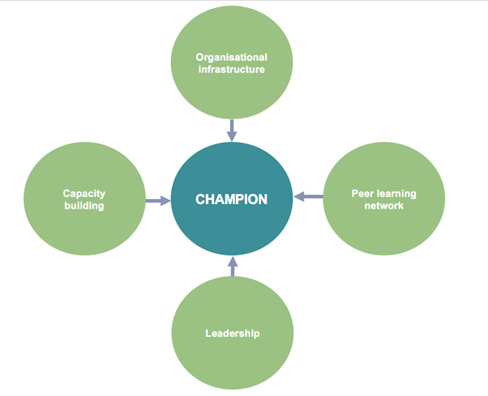
Components of the RESPIRE stakeholder engagement champion model.

From inception, RESPIRE was committed to working with a wide range of stakeholders to support locally relevant research and maximise the impact of the evidence generated. To achieve this goal, a dedicated platform was established to assist the LMICs research partners in implementing tailored engagement with stakeholders, such as patients, caregivers, members of the public, community leaders, frontline health workers, health care providers, policy makers, non-governmental organisations, clinical leaders and associations, and media agencies. Early on, the platform team observed challenges in applying the initial plans and training for stakeholder engagement, without a dedicated person in each partner organisation. Despite sharing broad principles and templates with all the partners, stakeholder engagement needed to be tailored to the unique contexts of each partner organisation and follow a locally driven approach. In response to these needs, the platform developed the stakeholder engagement champion model (**Figure 1**).

## STAKEHOLDER ENGAGEMENT CHAMPION MODEL

### Organisational infrastructure

A supportive organisational infrastructure was the foundation of the model (Figure S1 in the **Online Supplementary Document**). RESPIRE partners conducted respiratory research studies under two programmes (infectious and non-communicable diseases). They were guided by three governance units – an international steering committee (external experts), a unit management committee (with representatives from each country, programmes and platforms) and a directorate (for oversight and administration). The two research programmes were co-led by an expert from an LMIC partner country and the University of Edinburgh. These programmes and all the partners were supported by three platform teams: 1) community and stakeholder engagement, 2) capacity building, and 3) data science and methodology.

The community and stakeholder engagement platform comprised two expert co-leads and two postdoctoral research fellows (one experienced in engaging patients, public and community stakeholders and the other experienced in working with health system and non-state stakeholders). This platform oriented the partners to the role and process of stakeholder engagement in health research, supported them with stakeholder mapping and prioritisation tools, and offered technical advice and mentoring in planning and implementing engagement in their local contexts. Recognising the need for a designated person within each partner organisation, the platform collaborated with partners to design tailored role descriptions and recruit individuals familiar with the local context and stakeholders to undertake this role. Partners collectively decided to refer to this role as ‘stakeholder engagement champions.’ Champions worked closely with the researchers in their partner organisation to develop a contextualised plan, and lead the stakeholder engagement, thereby shifting the power and autonomy to make decisions around objectives, plans and resource allocation to the local partners.

Each country was allocated GBP 50 000 for engagement activities plus GBP 10 000 for the champion’s salary, with each champion’s salary receiving an additional GBP 2500 in the final year. Partners in each country jointly decided whether to appoint a single national champion to work across all organisations or to divide the GBP 10 000 funding to support one champion per partner organisation. Recruiting champions reflected the partners’ commitment to move beyond a ‘box-ticking’ exercise, towards more meaningful stakeholder engagement throughout their research studies. While the champion’s role was envisioned as a full-time job, partners adapted the working hours based on their needs and the time availability of the champion, as some had other responsibilities within the organisation.

### Features of a stakeholder engagement champion

Strong communication and interpersonal skills were prerequisites for the champions, both for engaging with internal stakeholders (*e.g.* principal investigators, research and organisational team) and external stakeholders (*e.g.* community, health care providers, policy makers). Champions had to understand stakeholder audiences, their needs and concerns, and be able to communicate relevant messages to diverse stakeholders, often with varying levels of power, interest, health literacy and research knowledge. Proficiency in local language(s) was essential to communicate with local and national stakeholders. Prior experience or familiarity with health research implementation and stakeholder engagement, either at the community or health-system level, was also required. Champions had to be well-versed in the local socio-economic, cultural, and political context, respiratory health challenges, and the affected communities. They could come from diverse educational backgrounds, as long as they demonstrated empathy, cultural sensitivity, a collaborative work ethic, willingness to learn, and a commitment to including marginalised or underserved stakeholders.

RESPIRE champions came from diverse professional backgrounds, including programme managers, social science and public health researchers, medical doctors, community health practitioners, and a retired public health programme director. Most champions were selected from the existing staff of partner organisations, which was advantageous given their familiarity with RESPIRE research plans, existing networks with stakeholders, and the potential to strengthen engagement capacity for future studies. In a few cases, champions were recruited externally. Over time, most champions recognised their role to garner buy-in for stakeholder engagement, especially within their own organisation, by sensitising researchers and senior leadership; the platform supported their efforts. A number of champions viewed this role as an opportunity to drive change in research and influence its impact.

### Capacity building

The platform’s research fellows organised monthly group meetings with the champions on Zoom (Zoom Communications, San Jose, California, USA). In these hour-long meetings, champions networked, exchanged field experiences, and attended talks by RESPIRE members and external experts from LMICs. The initial meetings oriented the champions to the fundamental concepts and principles, [14,15] while the talks presented practical examples of engagement, strategies for engaging community groups, underserved communities and individuals with low-literacy levels, working with media agencies, using social media and other platforms for dissemination; and approaches to sustain engagement during public health emergencies. There was an intention to bring all the champions together face-to-face to increase the sense of community and provide networking opportunities, but this was not possible due to the COVID-19 pandemic. The research fellows also conducted one-to-one meetings with each champion, providing tailored support in planning, implementing, and evaluating their community and stakeholder engagement activities.

An online repository was set up on Microsoft Teams (Microsoft, Redmond, Washington, USA) for the champions with access to relevant resources, including meeting minutes, published articles and grey reports. Champions were encouraged to disseminate their engagement work, and additional funding was provided for them to attend the 10th International Primary Care Respiratory Group world conference in 2021. Additional opportunities for accredited training were provided through funding champions to participate in the Stanford online course ‘Partnering with the Public and Patients in Medical Research’. One champion was supported in establishing a dedicated stakeholder engagement unit within their own research organisation, which aimed to work with stakeholders in all their future research studies as well as provide consulting services to other research and development organisations in the country.

### Peer network

Regular interactions among the champions facilitated a south-south exchange of experiences, ideas, solutions to challenges, and promoted experiential learning in community and stakeholder engagement. Together, the champions produced an animation explaining RESPIRE, translated into multiple languages to be shared via social media across the four partner countries. Champions sought feedback from this network regarding their engagement activities and outputs (*e.g.* a documentary film on patient and caregiver experiences living with pollen allergy in Pakistan) [16]. The champion from Malaysia shared their strategies for engaging young children and their parents in an asthma research study, including a comic activity book co-developed by young children and researchers to raise awareness about this health condition and promote its positive management in schools. This exchange prompted a champion from South India to consider creative ways to involve and educate young children in local communities about chronic respiratory health.

Champions used this forum to discuss both challenges and successes [17]. A champion from Pakistan shared their difficulties with study recruitment during the COVID-19 pandemic, seeking potential solutions from this peer network; while another champion from a western state in India detailed their intensive, yet successful community engagement efforts resulting in the recruitment of 14 500 participants for a sero-surveillance study [18]. Regular communication through a dedicated WhatsApp group fostered camaraderie and strengthened the bond among the champions.

### Leadership

This model encouraged champions to lead stakeholder engagement in their local sites and also develop resources based on their own field experiences and learning. Champions co-produced a resource guide [19] on how to plan, implement and evaluate stakeholder engagement in global health research complete with practical tools, illustrations and case studies from their own RESPIRE research projects. They also jointly published a peer-reviewed research article [17] that outlined the various strategies for sustaining stakeholder engagement during the COVID-19 pandemic, which was a feat in itself given the reported suspension of global health research [20].

Champions were supported to develop their independent research manuscripts and apply for additional funding to advance their engagement work. One champion was awarded an international fellowship in stakeholder engagement and also secured funding to engage marginalised and stateless communities in research aimed at improving access to tuberculosis-related health services in Malaysia. They also trained and mentored other researchers in their organisation to practise stakeholder engagement. Another champion was awarded a grant to engage female advocates in raising awareness around chronic respiratory diseases in rural communities in Pakistan.

## CHALLENGES

RESPIRE champions engaged stakeholders in both acute and respiratory health research, but found that achieving buy-in from decision-makers for interventions for chronic respiratory disease was slow and took time, because interest has necessarily focused on respiratory infection, which still remains a major cause of mortality in South Asia (even before COVID-19).

Sometimes, champions held limited power within their own organisation, which made decision-making challenging amidst hierarchical tensions. Champions who were new recruits or had less experience than other research team members needed some time to find their bearings and assert themselves when dealing with senior members in their organisation. The high-power distance culture [21] existing in several countries in Asia, including the RESPIRE network, makes it difficult to have an open discussion around power dynamics and ensure everyone is listened to regardless of their hierarchy in the organisation. Further, there are challenges of hierarchies and power in public health research and policy within each country, and unsurprisingly well-established research institutions have found it easier to build trusting relationships with influential stakeholders like policymakers. We tried to address this challenge by inviting national-level policymakers to a scientific meeting where all the partners got an opportunity to present evidence and actionable recommendations from their research. While this event sparked a dialogue between policymakers and partners, these presentations were still made by the senior researchers and not the champions.

Stakeholders may occasionally disagree with the engagement plan, or they might become disenchanted due to the whole process being time-consuming, the uncertainty of outcomes, or shifting priorities. Without the right experience, skills, and guidance, champions can find such situations demotivating and difficult to manage. If champions take on a part-time engagement role along with other research or project management tasks, then increased workloads or the lack of capacity to prioritise tasks can be taxing. Champions might also move on to newer roles, leading to staff turnover, and therefore affecting the difficult task of relationship building with stakeholders. This is challenging but can be addressed by investing in training all the research team members in stakeholder engagement processes, which can help fill the gaps and sustain engagement activities. Our learning has come through routine monitoring, observations, and discussions with the champions; we have not formally evaluated this model, which is a limitation. Future research is needed to explore the impact of this model, pathways for change as well as the barriers and facilitators.

## LESSONS LEARNED

Champion-led stakeholder engagement was one of the critical pathways that contributed to observed outcomes of RESPIRE, including patient and public involvement in research, raised community awareness, improved clinical practice, and policy influence [13]. Three important lessons emerge from our experience with this model. First, the champions were familiar with the context, challenges and research. Therefore, engagement was locally driven, and stakeholders were receptive to their efforts. Second, stakeholder engagement was decentralised, with champions and partner organisations deciding on the objectives, plans and resource allocation. Third, the engagement process undertaken by the champions was not a linear one; it was iterative. Champions learnt by ‘doing’, which involved making mistakes and learning from them, and this contributed to ownership and accountability for this work. Champions regularly communicated with stakeholders and periodically appraised and modified the extent of engagement based on the stakeholders’ levels of power and interest as well as the available resources. In this process, many champions built relationships with patient groups, primary health care providers and policy makers, fostering a sustainable dialogue beyond the research studies.

Successfully implementing this model in a global health research programme requires commitment from the senior leadership and institutionalisation in the form a dedicated policy, budget, and a supportive organisational infrastructure for the champions. The RESPIRE community and stakeholder engagement platform played a vital role in identifying, mentoring, and offering supportive supervision for the champions, while also facilitating and encouraging regular communication to nurture this fledgling community. This model also calls for flexibility to address any barriers, unexpected events, or crises. Contingency planning and subsequent reallocation of budget for stakeholder engagement activities during the COVID-19 pandemic are some examples of such flexibility. Apart from dedicated financial and technical support, non-monetary incentives such as training, skill-building, peer learning and support, co-production of resources, and recognition, were valuable for retaining champions’ interest and motivation.

Once institutionalised at an organisational level, champions can continue to engage with stakeholders even after the research project funding ends, as they will have forged links and relationships that can be sustained over time. Champions will need to be incentivised in appropriate ways, and external funding could be raised through local grants and the harmonisation of available resources.

## CONCLUSIONS

This model of a team of locally based champions can enable a community of practice of public health and development professionals who are interested in undertaking stakeholder engagement for translation and impact in global health research. Given the increasing calls for decolonising global health, our experiences present a practical step in this direction by shifting the traditional paradigm of knowledge and leadership and establishing a sustainable infrastructure through the stakeholder engagement champion model.

## Additional material


Online Supplementary Document
