# Supplementary material for: Stakeholder engagement champions: a locally-driven model for building impactful and sustainable relationships in global health research
Source: J Glob Health. 2025 Jun 6;15:03026. doi: 10.7189/jogh.15.03026 (PMC12143356; doi:10.7189/jogh.15.03026)

Supplement to: Jackson T, Fernandes G, Williams S, Nathan JJ, Makita M. Stakeholder engagement champions: a locally-driven model for building impactful and sustainable relationships in global health research. J Glob Health. 2025;15:03026.

Figure S1. RESPIRE's stakeholder engagement infrastructure.

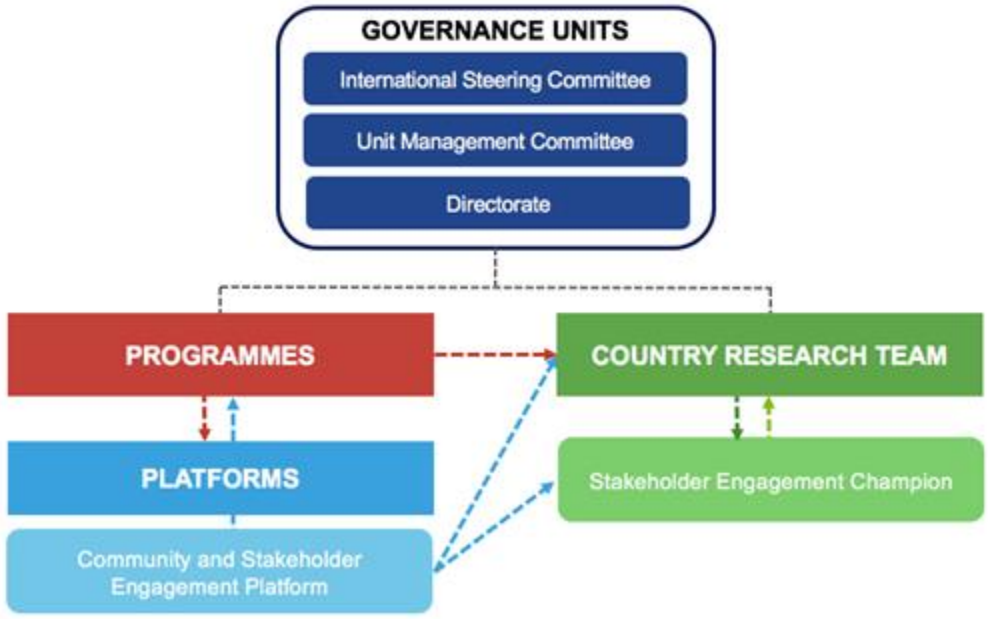

Supplement: Online Supplementary Document [file jogh-15-03026-s001.pdf]
